# Deployment Optimization Method of Multistatic Radar for Constructing Circular Barrier Coverage

**DOI:** 10.3390/s21196573

**Published:** 2021-09-30

**Authors:** Hai-Peng Li, Da-Zheng Feng, Shao-Feng Chen, Ya-Peng Zhou

**Affiliations:** 1 National Key Laboratory of Radar Signal Processing, Xidian University, Xi’an 710071, China; lihaipeng@stu.xidian.edu.cn; 2 Xi’an Electronic Engineering Research Institute, Xi’an 710100, China; zhou.yapeng@stu.xjtu.edu.cn; 3 Teaching and Research Support Center, Engineering University of PAP, Xi’an 710086, China; wjgcdxtsg@163.com

**Keywords:** multistatic radar, circular barrier coverage, deployment optimization, minimum deployment cost, deployment pattern

## Abstract

To construct circular barrier coverage (CBC) with multistatic radars, a deployment optimization method based on equipartition strategy is proposed in this paper. In the method, the whole circular area is divided into several sub-circles with equal width, and each sub-circle is blanketed by a sub-CBC that is built based on the multistatic radar deployment patterns. To determine the optimal deployment patterns for each sub-CBC, the optimization conditions are firstly studied. Then, to optimize the deployment of the whole circular area, a model based on minimum deployment cost is proposed, and the proposed model is divided into two sub-models to solve the optimization issue. In the inner model, it is assumed that the width of a sub-circle is given. Based on the optimization conditions of the deployment pattern, integer linear programming (ILP) and exhaustive method (EM) are jointly adopted to determine the types and numbers of deployment patterns. Moreover, a modified formula is introduced to calculate the maximum valid number of receivers in a pattern, thus narrowing the search scope of the EM. In the outer model, the width of a sub-circle is assumed to be a variable, and the EM is adopted to determine the minimum total deployment cost and the optimal deployment patterns on each sub-circle. Moreover, the improved formula is exploited to determine the range of width for a sub-circle barrier and reduce the search scope of the EM. Finally, simulations are conducted in different conditions to verify the effectiveness of the proposed method. The simulation results indicate that the proposed method can spend less deployment cost and deploy fewer transmitters than the state-of-the-artwork.

## 1. Introduction

In the last decades, the world witnessed the increasing popularity of wireless sensor networks (WSNs) because of their robust fault tolerance, high energy efficiency, strong data processing capability, and advanced wireless communication technology [1,2,3,4].

Barrier coverage is one of the essential applications of WSNs. It can be exploited to monitor the targets traversing through the boundary of an FoI [5]. To build an effective barrier coverage, the coverage area of the sensors should construct a continuous barrier covering the FoI. Compared with point coverage [6] and area coverage [7], barrier coverage obtains a balance between the number of sensors and the coverage area. Therefore, barrier coverage is efficient in monitoring intruders.

Most previous works on barrier coverage are based on the disk coverage model [8] or the sector coverage model [9]. The disk coverage model indicates that the coverage area is a circle with the sensor being located at the center, and the radius of the circle is the monitor range. While in the sector coverage model, the coverage area is a sector of the circle.

Recently, with the development of radar technologies, the multistatic radar based on a Cassini coverage model has drawn extensive attention [10,11]. Unlike the monostatic radar, which deploys a transmitter and a receiver at the same location, the multistatic radar is an important extension of bistatic radar. It consists of multiple transmitter-receiver pairs deployed at different locations [12]. The multistatic radar has several advantages, including strong anti-electronic jamming capacity, ease of anti-stealth aircraft, and ease of anti-low-altitude penetration [13,14,15]. The Cassini coverage model is derived from the bistatic radar equation. It is dependent on both the range between a pair of transmitter and receiver and the ranges from the target to the transmitter and receiver. Besides, two foci of Cassini coverage locate at a transmitter and a receiver [16].

Some works exploit the Cassini coverage model to build the line barrier coverage. In [17], the authors investigated the optimal deployment method for line barrier coverage to maximize the detectability of worst-case targets. In [18], a line barrier coverage consisting of heterogeneous transmitters was studied. The transmitters form different barrier coverages under different parameter settings. In [19], both the fault-tolerance issue and the energy-consumption issue of line barrier coverage were studied. The authors utilized the collaboration between transmitters and receivers on two consecutive deployment lines to obtain a minimum deployment cost.

Other works focus on building circular barrier coverage (CBC) to detect the invader who tries to enter the closed area. CBC can be exploited in various scenarios, such as monitoring coastlines, contaminated zones, and international boundaries. In these scenarios, the protected area has an irregular shape, which can be approximated by its minimum circumcircle and several multistatic radars are deployed in this circle. Thus, it is necessary to build circle barrier coverage on the circular FoI [20]. However, due to the properties of the Cassini coverage model, it is challenging to optimize the deployment of multistatic radars to form the circle barrier coverage. Meanwhile, to ensure the monitor effectiveness, it is desirable to set a minimum threshold for the width of circle barrier coverage. Besides, to achieve an excellent and cost-efficient circle barrier coverage, the unit costs of the transmitter and receiver should be considered by the optimization model.

To overcome these challenges, this paper uses multistatic radars to construct CBC and develops an improved optimization method based on the equipartition strategy. First, the optimization conditions of CBC are proven through analyzing the properties of multistatic radar deployment patterns (simply referred to as “deployment patterns” in the subsequent content). Based on these conditions, given the number of transmitters and receivers, the deployment pattern for obtaining a maximum coverage area can be determined. Moreover, the transmitter-receiver cost ratio is considered to derive the optimal deployment with a minimum cost. Besides, to optimize the deployment on the whole circular area, a two-stage optimization algorithm is proposed. Specifically, in the first stage, this area is divided into several sub-circles with equal width. Thus, the total deployment cost is equal to the sum of each sub-circle barrier cost. In the second stage, since the width of a sub-circle is already determined in the first stage, integer linear programming (ILP) [21] and exhaustive method (EM) are adopted to jointly determine the types and numbers of deployment patterns in the given sub-circle barrier. Finally, the optimization results in the condition of minimum cost can be obtained, and the proposed method is verified by the simulation results.

The main contributions in this paper are summarized as follows:

First, the properties of deployment patterns are studied, and the conditions for achieving the optimal deployment patterns in a circular barrier are proven. Based on these conditions, the optimization model for the whole circular FoI is introduced. Meanwhile, this model is divided into two sub-optimization models to obtain the solution of this model.

Secondly, a novel formula is proposed to calculate the maximum valid number of receivers in a deployment pattern. Moreover, another novel formula is introduced to calculate the upper threshold of width for a sub-circular barrier. These two formulas can dramatically reduce the computation.

Thirdly, a two-stage optimization algorithm with an equipartition strategy is proposed to solve these two sub-optimization models. In the condition of minimum deployment cost, the optimal deployment is derived by considering the transmitter-receiver cost ratio. The simulation results indicate that the proposed method needs less deployment cost and requires fewer transmitters than the works in the literature.

The rest of this paper is organized as follows. Section 2 reviews the related work. Section 3 introduces the concepts and details of the optimization issue. Section 4 proposes the solution for the optimization issue. Section 5 conducts the simulations to validate the performance of the proposed method. Section 6 concludes the paper.

## 2. Related Work

Regarding various scenarios and tasks, barrier coverage problems were extensively and profoundly studied in the last decade. The models that have been studied can be divided into three categories: the disk coverage model [8,22,23,24,25], sector coverage model [9,26,27,28,29], and Cassini coverage model [20,30,31,32,33].

In the beginning, the works on the disk coverage model were reviewed. In [8], the notion of weak and strong barrier coverage was introduced, and a critical condition was derived for constructing the weak barrier coverage. In [22], for the barrier coverage composed of a set of wireless sensors with adjustable ranges, the constant-approximation algorithms were designed to assign the ranges with the minimum cost, and the constant depends on the covering radius related to the power of sensors. In [23], crossed barrier coverage was built to detect the intrusion behavior in both horizontal and vertical directions. Meanwhile, the branch-and-bound algorithm and multi-round shortest path algorithm are exploited to determine the maximum number of crossed barriers. Given a set of mobile sensors distributed on the plane and a set of targets located on a line, an exact algorithm was introduced to minimize the maximum movement of the sensors the work and cover the targets with minimum energy consumption [24]. In [25], the inspection robot was integrated into WSNs to form an efficient dynamic barrier coverage for monitoring the transmission lines. Moreover, a discrete heuristic method based on game theory was proposed to solve this issue.

Considering the works based on the sector coverage model, in [9], sensors with arbitrarily adjustable directions were exploited to construct the strong barrier coverage. To determine the appropriate directions of sensors in the one-dimension scenario, a polynomial-time algorithm was adopted to achieve strong barrier coverage with a minimum number of sensors. Meanwhile, in the two-dimension scenario, the directional carrier graph was introduced to solve the issue. In [26], the researchers surveyed the concepts and properties of the sector coverage model, and they analyzed the sensing characters and behaviors of directional sensors. Besides, each category of the sector coverage model was summarized in terms of issue description, assumption, application, solution, and performance. The researchers in [27] addressed the barrier gap problem of weak and strong barrier coverage and deployed the directional sensors with a line deployment strategy. Besides, they proposed a gap-finding algorithm to determine sub-barriers and locate barrier gaps, and they fixed the gaps through two gap-mending algorithms. In [28], the sector coverage model was applied in a three-dimension scenario, and a realistic resolution criterion of the camera sensor was proposed to capture the face characters of the invader. Based on this criterion, a feasible deployment strategy was studied for barrier coverage. In [29], the virtual target–barrier construction method was adopted to construct a closed circular belt around a target. The closed virtual barrier curve was exploited to simplify the optimization problem, and then a distributed particle swarm optimization algorithm was designed to deploy an approximate optimal coverage on the virtual barrier curve.

There is increasing attention on the Cassini coverage model. In [30], given the numbers of transmitters and receivers, an optimal linear barrier coverage was constructed. However, this barrier coverage does not have a requirement of minimum width. This means that in some parts, the barrier is only covered by some points, and a high-speed intruder can cross the barrier without detection. The authors in [31] stated that it is optimal to place multistatic radars on the shortest deployment line connecting the left and right boundaries of the region. Based on these achievements, an optimization deployment method was proposed to build barrier coverage on the rectangular area [32]. Specifically, the method divides the whole rectangular area into multiple sub-rectangles with equal width. Then, it sets the minimum width for a sub-rectangle. Finally, several identical deployment patterns are exploited to build barrier coverage for each sub-rectangle.

Besides, some works focus on constructing an optimal barrier coverage on a circular area, which is a challenging issue due to the circular shape. As for this shape, the width and radius of a sub-circular barrier should be considered. An optimal model was introduced to build one CBC [20]. Practically, one circular barrier is inadequate to cover a vast circular area, and the width of a circular barrier is not set with a threshold. A model with a threshold of width was mentioned in [33], and the whole circular area was divided into several sub-circular barriers with the same width. Each sub-circular barrier was composed of several identical deployment patterns, and only a varied one was exploited to cover the remaining part. Besides, EM was adopted to determine the optimal results. However, the composition of a sub-circular barrier and the searching scope of EM can be further improved. Inspirited by [33], our work makes the following improvements: (1) several novel conditions are introduced to determine the types of deployment patterns for an optimal sub-circular barrier; (2) ILP is adopted to obtain the corresponding number of deployment patterns; (3) an improved formula is introduced to calculate the maximum valid number of receivers in a deployment pattern; (4) a novel formula is adopted to calculate the upper threshold of width to narrow the searching scope of sub-circular barrier width.

## 3. Conceptions and Problem Descriptions

### 3.1. Conceptions of Multistatic Radar Coverage

In this paper, the deployment pattern of multistatic radar is composed of a transmitter T and several receivers $R_{i},$ (i=1,2,…,m), and it can be denoted as $F^{m}=\left({T,R}_{1},\ldots {,R}_{m}\right)$. Thus, the multistatic radar can be regarded as a combination of multiple bistatic radars that share a transmitter. In terms of coverage area, it is desirable to use the bistatic radar to illustrate the coverage properties of the multistatic radar.

Taking $F^{1}$ as an example, the signal-to-noise ratio (SNR) at point A, i.e., SNR(A), can be computed by the radar equation of coherent pulse bistatic radar [16], shown as follows:

(1) $$SNR(A)=K_{b}/{\left(d_{T}(A)d_{R_{1}}(A)\right)}^{2}$$

where $K_{b}$ is assumed to be a constant determined by the working parameters of the bistatic radar [32]. Let $d_{T}\left(A\right)$ and $d_{R_{1}}\left(A\right)$ represent the distance from point A to the transmitter and the receiver, respectively.

Let γ represent the minimum SNR that is the threshold for target detection. If $SNR\left(A\right)\geq \gamma$, the target is in the coverage area of a bistatic radar; otherwise, the target is out of the coverage area. Therefore, the coverage area of the bistatic radar is defined by the formula $d_{T}(A)d_{R}(A)\leq$ $\sqrt{K_{b}/\gamma }$. For ease of presentation, we have $l_{\max}=\sqrt[{4}]{K_{b}/\gamma }$. It is noteworthy that a target in the coverage area can be detected with an acceptable probability [34].

**Figure 1:**
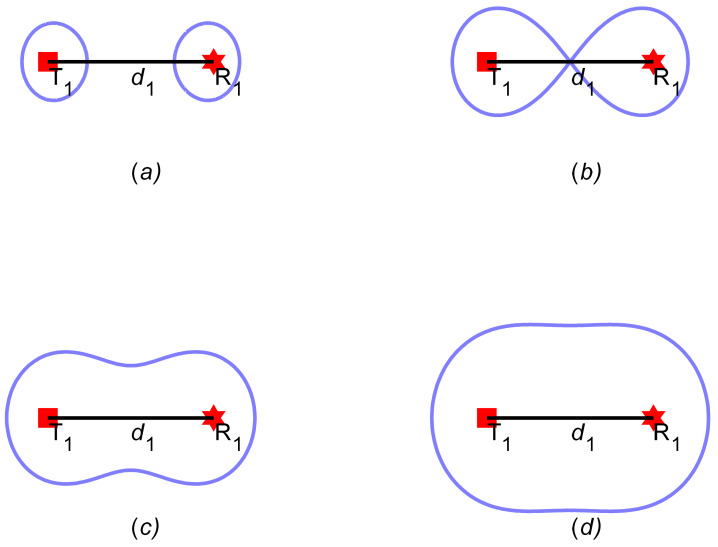
The coverage area of bistatic radar under different parameters; (**a**) $d_{1}>l_{\max}$; (**b**) $d_{1}=l_{\max}$; (**c**) $\sqrt{2}l_{\max}/2\leq d_{1}<l_{\max}$; (**d**) $0<d_{1}<\sqrt{2}l_{\max}/2$.

According to [16], the shape of the coverage area is determined by the relation between $d_{1}$ and $l_{\max}$, as shown in Figure 1:

- $d_{1}>l_{\max}$, the coverage is two unconnected areas, as plotted in Figure 1a;
- $d_{1}=l_{\max}$, the coverage is enclosed by a lemniscate of Bernoulli, as plotted in Figure 1b;
- $\sqrt{2}l_{\max}/2\leq d_{1}<l_{\max}$, the coverage is enclosed by a waist-liked area, as plotted in Figure 1c;
- $0<d_{1}<\sqrt{2}l_{\max}/2$, the coverage is enclosed by an elliptical curve, as plotted in Figure 1d.

It is also assumed that transmitters work at orthogonal frequencies. By choosing a working frequency, a receiver can pair with a certain transmitter and avoid being interfered with by other transmitters. Thus, the coverage area of the multistatic radar $F^{m}$ is overlapped with that of *m* bistatic radars. If a target is covered by multiple bistatic radars, the maximum SNR at point A, i.e., $SNR_{\max}(A)$, is exploited to measure the coverage of the multistatic radar, which is formulated as:

(2) $$SNR_{\max}(A)=K_{b}/\underset{\left(T_{j},R_{i}\right)}{\min}l{\left(A\right)}^{2}$$

where $l\left(A\right)=d_{T_{j}}(A)d_{R_{i}}(A)$, $T_{j}$ denotes the *j*th transmitter. Based on this, for a target at arbitrary point A, it can be covered by the multistatic radar if $SNR_{\max}(A)\geq \gamma$, and we have $l\left(A\right)\leq l_{max}^{2}$

### 3.2. Conceptions of CBC

**Figure 2:**
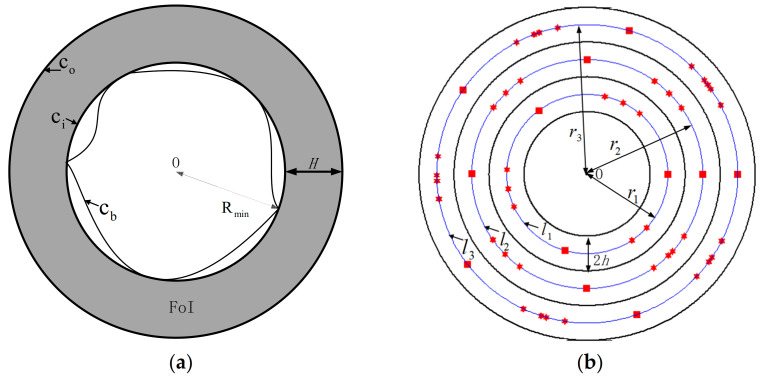
(**a**) The enclosed area is rounded by a circular FoI with a width of *H* and an inner radius of *R*_min_. (**b**) An FoI is divided into three sub-circles, where the black curve represents the boundary of a sub-circle and the blue curve represents the deployment curve.

As for a closed area with irregular boundaries, it can be represented by a minimum circumscribed circle, and a circular FoI is built around the circle to protect the closed area from infiltrating. As shown in Figure 2a, the gray circle area represents the circular FoI with a width of *H*, and the center of the FoI is O. $c_{i}$ and $c_{o}$ respectively denote the inner and outer circle boundary of the FoI. Besides, $R_{\min}$ represents the radius ***c****_i_*.

In this paper, the whole FoI is divided into multiple sub-circles with equal width, and each sub-circle is covered by a sub-CBC. By constructing a sub-CBC with a width of 2 *h*, multiple multistatic radars are deployed along the ring in the middle of a sub-circle. This ring is called the deployment curve, and it is denoted as $l_{k},\;k=1,\ldots ,q$. The radius of $l_{k},k=1,\ldots ,q$ is denoted as $r_{k}$. Here, 2h≥2η>0, where 2η is the minimum width threshold of a sub-circle, and 2 *h* is the minimum width of a sub-CBC.

For instance, an FoI is divided into three sub-circles in Figure 2b. Without loss of generality, all the sub-CBCs are numbered from inside to outside. Correspondingly, $l_{k},\;k=1,2,3$ represents the deployment curve of each sub-CBC. Moreover, the nodes are deployed in counterclockwise order. The transmitter is represented by a square, and the receiver is represented by a hexagram.

It is desirable to study the deployment optimization for multistatic radars on a deployment curve. For ease of presentation, two definitions are given:

**Definition** **1.** *Let*$P_{i}^{n}$*denote a deployment pattern comprised of n receivers and two transmitters, i.e.,*$P_{i}^{n}=\left(T_{i},R_{1},\ldots ,R_{n},T_{i+1}\right)$*. Without ambiguity, it can be denoted as*$P^{n}$*for short. Note that n receivers are deployed between two transmitters, i.e.,*$T_{i}$*and*$T_{i+1}$*. Thenodecoverage angle (NCA) in*$P_{i}^{n}$*is denotedas*$2\theta_{j},\;j=1,\ldots ,n+1$*, where*$2\theta_{1}=∠T_{i}OR_{1}$*,*$2\theta_{j}=∠R_{j-1}OR_{j}$*,*j=2,…,n*,*$2\theta_{n+1}=∠R_{n}OT_{i+1}$.

According to [33], NCA can be calculated as:

(3) $$\begin{array}{l} \theta_{k}=\left\{\begin{array}{l} \mathrm{acos}(\frac{r^{2}+R^{2}-l_{\max}^{2}}{2rR}),\;k=1\\ \mathrm{acos}\left\{\frac{\left(r^{2}+R^{2})\cos(\sum_{m=1}^{k-1}\theta_{m}\right)}{2rR}-\frac{\sqrt{{\left(R^{2}-r^{2}\right)}^{2}(\cos^{2}(\sum_{m=1}^{k-1}\theta_{m})-1)+l_{\max}^{4}})}{2rR}\right\}\\ -\sum_{m=1}^{k-1}\theta_{m},\;\;\;\;\;\;\;\;\;\;\;\;\;\;\;\;\;k=2,\ldots ,\left\lceil \left(n+1\right)/2\right\rceil \end{array}\right.\\ \theta_{k}=\theta_{n-k+2},k=1,\ldots ,\left\lceil \left(n+1\right)/2\right\rceil \end{array}$$

where, *r* denotes the radius of the deployment curve *l*, and *R* denotes the radius of the outer boundary of the sub-circle. Besides, $R_{j}$ is a valid receiver if $\theta_{j}>0,\;j=1,2,\ldots ,n+1$.

Furthermore, according to Formula (3), by using algebraic operation, we have

(4) $$\sum_{m=1}^{k}\theta_{m}=\mathrm{acos}\left\{[(r^{2}+R^{2})\cos(\sum_{m=1}^{k-1}\theta_{m})-\sqrt{{\left(R^{2}-r^{2}\right)}^{2}(\cos^{2}(\sum_{m=1}^{k-1}\theta_{m})-1)+l_{\max}^{4}})]/2rR\right\}$$

Let $\omega_{P}(n,h,r)$ denote the central angle formed by $P^{n}$, and it is referred to as pattern coverage angle (PCA). Usually, a PCA is inadequate to cover the whole FoI. Thus, we have $\omega_{P}(n,h,r)=∠T_{i+1}OT_{i}=\sum_{j=1}^{n+1}2\theta_{j}<360^{\circ }$. $\omega_{P}(n,h,r)$ can be calculated as follows:

(5) $$\omega_{P}(n,h,r)=\left\{\begin{array}{ll} 4\sum_{k=1}^{\frac{n}{2}}\theta_{k}+2\theta_{\frac{n}{2}+1}, & \mathrm{if}\;n\;\mathrm{is}\;\mathrm{an}\;\mathrm{even}.\\ 4\sum_{k=1}^{\frac{n+1}{2}}\theta_{k}, & \mathrm{if}\;n\;\mathrm{is}\;\;\mathrm{an}\;\mathrm{odd}. \end{array}\right.$$

**Definition** **2.** *Let* S*denote a deployment sequence formed by* k *varied types of*$P^{n_{j}},\;j=1,2,\ldots ,k$*. Let*$s_{j},\;j=1,2,\ldots ,k$*denote the number of types. Specifically, we have*$S=\{s_{1},P_{1}^{n_{1}},\ldots ,s_{k},P_{k^{\prime }}^{n_{k}}\},\;k^{\prime }=\sum_{j=1}^{k-1}s_{j}+1$. *To build a CBC,*$\sum_{j=1}^{k}s_{j}\omega_{P_{n_{j}}}(n_{j},h,r)\geq 360^{\circ }$*should hold.*

**Figure 3:**
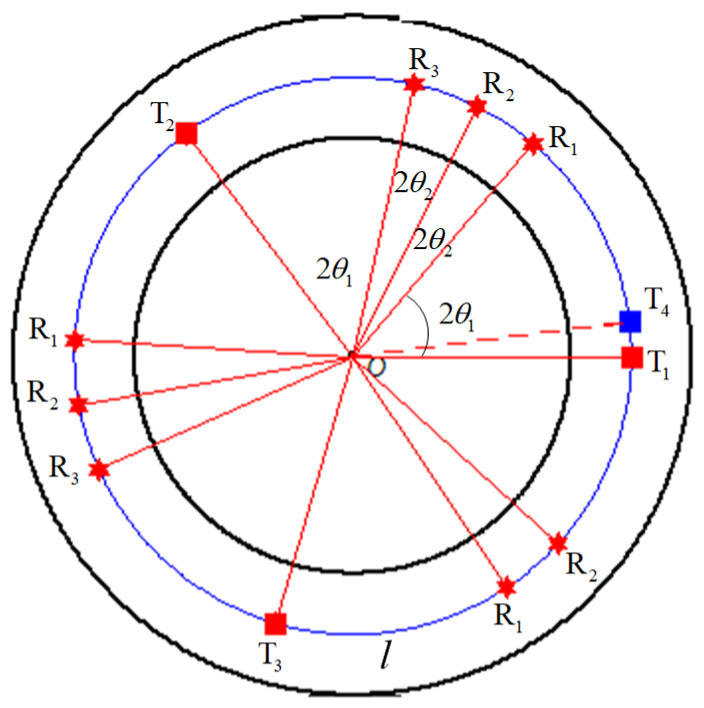
Deployment diagram for the deployment sequence $S=\left\{2{,P}_{1}^{3}{,1,P}_{3}^{2}\right\}$.

As shown in Figure 3, in the case where $r=3.833\;\mathrm{km},\;l_{\max}=2\;\mathrm{km},\;R=4.667\;\mathrm{km}$, there is a sub-circle covered by the deployment sequence of $P_{1}^{3},P_{2}^{3},P_{3}^{2}$, and all patterns are deployed on the deployment curve *l*. According to (5), the total angle formed by these three patterns equals to 367.2°, which is a bit larger than 360°. In theoretical, the transmitter T_4_ should be placed where the blue square is plotted. However, in practice, $P_{1}^{3},P_{3}^{2}$ share the transmitter T_1_.

### 3.3. Description of Optimization Deployment Problem

The problem can be described as follows. Let D denote the FoI, and FoI is equally divided into *q* sub-circles with a width of 2 *h*. The CBC is exploited to cover each sub-circle. Let $M_{k}$ and $N_{k},\;(k=1,\ldots ,q)$ denote the number of transmitters and receivers on each deployment curve, respectively. Besides, it is assumed that all the transmitters are identical, so are the receivers. In addition, the unit costs of the transmitters and receivers are denoted as $c_{T}$ and $c_{R}$, respectively. Let $\alpha =c_{T}/c_{R}$ denote the transmitter-receiver cost ratio, and it is reasonable to set α>1.

Under the minimum deployment cost, $c_{\mathrm{tot}}$, the optimization problem model can be formulated as follows:

(6) $$\begin{array}{l} \mathrm{minimize}\;c_{\mathrm{tot}}=\sum_{k=1}^{q}\left(M_{k}c_{T}+N_{k}c_{R}\right)\\ s.t.\;\;l(A)\leq l_{\max}^{2},\;\forall A\in D \end{array}$$

In this paper, $\left\lfloor k\right\rfloor$ rounds *k* to the nearest integer towards minus infinity; $\left\lfloor k\right\rfloor$ rounds *k* to the nearest integer towards positive infinity; Φ denotes an empty set; $N^{+}$ and $N^{*}$ denote the positive integer set and natural integer set, respectively. For point A and A′, $\left|AA\prime \right|$ denotes the length of the line AA′.

## 4. Solution for Optimization Deployment Problem

### 4.1. The Upper Threshold of Width for a Sub-Circular Barrier

As mentioned above, 2μ is the lower threshold width of the CBC. In this section, a novel formula is introduced to determine the upper threshold width of the CBC. Like the conditions in [33], the waist-liked area in the bistatic radar is exploited first. Then, Lemma 1 is adopted to determine $h_{\sup}$.

**Lemma** **1.** *As for the width-equipartition strategy, $h_{\sup}$ denotes the upper threshold of h, and it can be calculated as follows:*

(7) $$h_{\sup}=\frac{1}{3}(\sqrt{2}l_{\max}+\sqrt{R_{\min}^{2}+\sqrt{2}R_{\min}l_{\max}-l_{\max}^{2}}-R_{\min})$$

**Proof of** **Lemma 1.** The proof of Lemma 1 is given in the Appendix A.  □

According to Formula (3), we have $l_{\max}^{}-2r\leq h\leq l_{\max}^{}$. Thus, the range of barrier width can be modified to $2\max\{l_{\max}^{}-2r,\eta \}<2h\leq 2h_{\sup}^{}$. Subsequently, let *q* denotes the number of sub-circles. According to the equipartition strategy, $q=\left\lceil H/2h\right\rceil$. Thus, we have $q_{\max}=\left\lceil H/\left(2\max\{l_{\max}^{}-2r,\eta \}\right)\right\rceil$ and $q_{\min}=\left\lceil H/2h_{\sup}\right\rceil$.

### 4.2. The Maximum Number of Receivers and the Property of NCA

In this section, Lemma 2 is introduced to improve the formula for determining the maximum number of receivers in a $P^{n}$. Then, Lemma 3 is exploited to uncover the property of NCA.

**Lemma** **2.** *Let 2 h denote the width of a sub-circular barrier, and r denote the radius of the deployment curve. Let $n_{\max}$ denote the maximum number of receivers in $P^{n}$. Then $n_{\max}$ is calculated as follows:*

*(1) If $h_{\sup}^{}\geq$ $h>\sqrt{r^{2}+l_{\max}^{2}}-r$ and u is the largest natural number that satisfies the inequality:*

$$\sum_{m=1}^{u}\theta_{m}\leq \mathrm{acos}\left(\sqrt{\frac{{\left(2r+h\right)}^{2}h^{2}-l_{\max}^{4}}{4r(r+h)h^{2}}}\right)$$

*then $n_{\max}=2u+1$ and $2\Sigma_{m=1}^{u+1}\theta_{m}<180^{0}$.*

*(2) If $\max\{l_{\max}^{}-2r,0\}<h\leq \min\{\sqrt{r^{2}+l_{\max}^{2}}-r,h_{\sup}\}$ and u is the largest natural number that satisfies the inequality:*

$$\sum_{m=1}^{u}\theta_{m}\leq \mathrm{acos}\left(\sqrt{\frac{l_{\max}^{4}-{\left(2r+h\right)}^{2}h^{2}}{4r^{2}{\left(r+h\right)}^{2}}}\right)$$

*then $n_{\max}=2\left(u+1\right)$.*

**Proof of** **Lemma 2.** The proof of Lemma 2 is given in the Appendix A.  □

By contrast, it is claimed in [33] that $\theta_{\max}=\mathrm{acos}(\frac{(R^{2}+r^{2}){\left(R-r\right)}^{2}-l_{\max}^{2}}{2rR{\left(R-r\right)}^{2}})$. However, this formula is ineffective in some conditions. To prove this, given $r=4\;\mathrm{km},\;R=4.3\;\mathrm{km},\;l_{\max}^{}=\sqrt{3}\;\mathrm{km}$, based on [33], we have $\theta_{\max}=\mathrm{acos}(-1.9044)$.

According to Formula (5), the more receivers are placed in $P^{n}$, the larger a central angle can be formed. However, Lemma 3 states that the efficiency of placing receivers decreases as the number of receivers increases.

**Lemma** **3.** *For $P^{n}$ it is assumed that $\sum_{k=1}^{u}\theta_{k}<\varphi$, $u=\left\lceil \left(n+1\right)/2\right\rceil$. Then, the NCA sequence $\theta_{k},\;k=1,2,\ldots ,u$ is strictly monotonous decreasing. Where,*

$$\varphi =\left\{\begin{array}{l} \mathrm{acos}(\sqrt{\frac{{\left(2r+h\right)}^{2}h^{2}-l_{\max}^{4}}{4r(r+h)h^{2}}}),\;h>\sqrt{r^{2}+l_{\max}^{2}}-r\\ \mathrm{acos}(\sqrt{\frac{l_{\max}^{4}-{\left(2r+h\right)}^{2}h^{2}}{4r^{2}{\left(r+h\right)}^{2}}}),\;0<h\leq \sqrt{r^{2}+l_{\max}^{2}}-r \end{array}\right.$$

**Proof of** **Lemma 3.** The proof of Lemma 3 is given in the Appendix A.  □

For $P^{n}$, let $\lambda_{j}=\theta_{j}/c_{R}$ denote the effectiveness-cost ratio (ECR) of $R_{j},\;j=1,2,\ldots ,n$. It reveals the coverage efficiency of a receiver. The larger $\lambda_{j}$, the better coverage efficiency of a receiver. Besides, let $\bar{\lambda }=\frac{1}{n}\sum_{j=1}^{n}\lambda_{j}$ denote the average ECR of $P^{n}$, and it reveals the coverage efficiency of $P^{n}$.

Moreover, Lemma 3 states that $\lambda_{j}$ decreases as j increases. In other words, the farther a receiver is placed away from the transmitter, the lower ECR is obtained. Meanwhile, the larger *n*, the lower average ECR of $P^{n}$.

### 4.3. The Conditions of the Optimal Deployment Patterns for a CBC

According to the equipartition strategy, it is critical to find the optimization conditions of deployment patterns on a sub-CBC. These conditions are essential to establishing the optimization model of the deployment problem.

Given h and r, for ease of presentation, $\omega_{P}(n,h,r)$ can be abbreviated as $\omega_{P}(n)$.

**Lemma** **4.** *Assuming two varied patterns $P^{n_{1}}$ and $P^{n_{2}}$ in the CBC and $\max\{n_{1},n_{2}\}\leq n_{\max}$, the following statements holds.*

*(1) If $n_{1}+n_{2}=2v$, $\omega_{P}(n_{1})+\omega_{P}(n_{2})\leq 2\omega_{P}(v)$ holds if and only if $n_{1},n_{2}$ are equal or $n_{1},n_{2}$ are consecutive odd;*

*(2) If $n_{1}+n_{2}=2v+1$, $\omega_{P}(n_{1})+\omega_{P}(n_{2})\leq \omega_{P}(v)+\omega_{P}(v+1)$ holds if, and only if $|n_{1}-n_{2}|=1$.*

**Proof of** **Lemma 4.** The proof of Lemma 4 is given in the Appendix A.  □

Lemma 4 provides the necessary and sufficient conditions for an optimal CBC. Moreover, it reveals that the PCA of $P^{2n-1}$ and $P^{2n+1}$ can be equally replaced by two PCAs of $P^{2n}$. Thus, *P*^2*n*−1^, *P*^2*n*^, and *P*^2*n*+1^ can be replaced with three *P*^2*n*^ to simplify the structure of the deployment sequence and make it easier for engineering. Furthermore, due to $\omega_{P}(2n)+\omega_{P}(2n+2)<2\omega_{P}(2n+1)$, it is necessary to deploy three *P*^2*n*+1^ instead of *P*^2*n*^, *P*^2*n*^*^+^*^2^, and *P*^2*n*+1^ in an optimal CBC. Supposing that $n_{1}=2,\;n_{2}=4,\;r=4\;\mathrm{km},R=4.8\;\mathrm{km},$ $\;l_{\max}^{}=\sqrt{3}\;\mathrm{km}$, according to [33], the coverage angle is $\omega_{P}(2)+\omega_{P}(4)=188.67^{\circ }$. By contrast, based on Lemma 4, we have $2\omega_{P}(3)=197.75^{\circ }$. It means that the proposed conditions can contribute to a better optimization result.

### 4.4. Model and Solution for Deployment Optimization Problem

It is revealed by Lemma 4 that, for a deployment sequence, the maximum coverage angle can be achieved by using two varied patterns at most. Moreover, the difference in the number of receivers in patterns should not be larger than 1. This deployment sequence is referred to as the optimal one. Besides, it is assumed that the whole circular area is equally divided into *q* sub-circular barriers. Let $S_{\mathrm{opt}}^{k}=\left\{s_{1}^{k},P^{n_{k}},s_{2}^{k},P^{n_{k}+1}\right\},\;1\leq k\leq q$ denote the optimal deployment sequence for the *k*th sub-circular barrier. Without loss of generality, k=1 represents the innermost sub-circular barrier and k=q represents the outmost sub-circular barrier. Then, the deployment optimization model can be represented as follows:

(8) $$\begin{array}{l} \underset{\left\{q,s_{1}^{k},s_{2}^{k},n_{k}\right\}}{\min}\;c_{\mathrm{tot}}=\sum_{k=1}^{q}\left\{s_{1}^{k}(n_{k}+\alpha )+s_{2}^{k}(n_{k}+1+\alpha \right)\}\\ \;\;\;s_{1}^{k}\omega_{P}(n_{k},h_{q},r_{k})+s_{2}^{k}\omega_{P}(n_{k}+1,h_{q},r_{k})\geq 2\pi \\ s.t.\;n_{k}\leq n_{\max}^{k},\;\;(k=1,2,\ldots ,q),\;H/q=2h_{q},\;s_{i}^{k}\in N^{0},\\ n_{k}\in N^{*},\;r_{k}=R_{\min}+(2k-1)h_{q},\;q_{\min}\leq q\leq q_{\max}\; \end{array}$$

However, the nonlinearity and multiple parameters make the solution to this model difficult. To overcome these obstacles, the solution to this model is decomposed to a two-stage optimization problem:

$\underset{\left\{q,s_{1}^{k},s_{2}^{k},n_{k}\right\}}{\min}c_{\mathrm{tot}}=\underset{q}{\min}\{\sum_{k=1}^{q}\underset{\left\{s_{1}^{k},s_{2}^{k},n_{k}\right\}}{\min}c_{s}^{k}\}$, where $c_{s}^{k}$ denotes the minimum deployment cost for the *k*th sub-CBC. In this stage, it is assumed that the width of each sub-circle is given. Then, $q_{\min}$ and $q_{\max}$ can be calculated. For each $q,\;(q_{\min}\leq q\leq q_{\max})$ and $q\in N^{+}$, $2h_{q}=H/q$, the following sub-optimization problem is solved:

(9) $$\begin{array}{l} \underset{\left\{s_{1}^{k},s_{2}^{k},n_{k}\right\}}{\min}c_{s}^{k}=s_{1}^{k}(n_{k}+\alpha )+s_{2}^{k}(n_{k}+1+\alpha )\\ s.t.\;s_{1}^{k}\omega_{P}(n_{k},h_{q},r_{k})+s_{2}^{k}\omega_{P}(n_{k}+1,h_{q},r_{k})\geq 2\pi \;\\ s_{i}^{k}\in N^{0},\;n_{k}\in N^{*},\;r_{k}=R_{\min}+(2k-1)h_{q},\;n_{k}\leq n_{\max}^{k},\;k=1,2,\ldots ,q \end{array}$$

For each sub-CBC, there is a model depicted by Formula (9).

$\underset{\left\{s_{1}^{k},s_{2}^{k},n\right\}}{\min}c_{s}^{k}=\underset{n\leq n_{\max}^{k}}{\min}\{\underset{\left\{s_{1}^{k},s_{2}^{k}\right\}}{\min}c_{s}^{k,n}\}$, where $c_{s}^{k,n}=s_{1}^{k}(n+\alpha )+s_{2}^{k}(n+1+\alpha )$. In this stage, supposing that *n* is determined, $c_{s}^{k,n}$ denotes the deployment cost of the *k*th sub-CBC in this condition. *n* is the number of receivers in a pattern. Besides, based on Lemma 2, the maximum number of receivers $n_{\max}^{k}$ for the patterns in the *k*th sub-CBC can be calculated. The following sub-optimization problem can be solved:

(10) $$\begin{array}{l} \underset{\left\{s_{1}^{k},s_{2}^{k}\right\}}{\min}c_{s}^{k,n}=s_{1}^{k}(n+\alpha )+s_{2}^{k}(n+1+\alpha )\\ s.t.\;\;\;s_{1}^{k}\omega_{P}(n,h_{q},r_{k})+s_{2}^{k}\omega_{P}(n+1,h_{q},r_{k})\geq 2\pi ,\;s_{i}^{k}\in N^{0} \end{array}$$

Formula (10) can be solved by using the ILP.

The specific process of solving the deployment optimization model is given:

1: Calculate $h_{\sup}$, $\;q_{\min}$ and $\;q_{\max}$ through Lemma 1. Initialize the deployment curve set Q=Φ and the total deployment cost set $C_{\mathrm{tot}}=\Phi$
2: For $q=q_{\min}:q_{\max}$
3: Calculate h=H/2q.
4: For  k=1:q, calculate $n_{\max}^{k}$
5: For $\;n=1:n_{\max}^{k}$
  Based on Formula (10), the cost $c_{s}^{k,n}$ is obtained.
6: end for
7: $c_{s}^{k}\leftarrow \min\left\{c_{s}^{k,1},c_{s}^{k,2},\ldots ,c_{s}^{k,n_{\max}^{k}}\right\}$, as depicted by the sub-optimization problem ②
8: end for
9: $c_{\mathrm{tot}}^{q}\leftarrow \sum_{k=1}^{q}c_{s}^{k}$. Let $c_{\mathrm{tot}}^{q}$ denote the minimum deployment cost in the case where the whole area is divided into *q* sub-circles. Then, $Q=Q\cup \left\{q\right\},\;C_{\mathrm{tot}}=C_{\mathrm{tot}}\cup \left\{c_{\mathrm{tot}}^{q}\right\}$.
10: end for
11: $c_{\mathrm{tot}}^{*}\leftarrow \min C_{\mathrm{tot}}$, as depicted by the sub-optimization problem ①
  $index=C_{\mathrm{tot}}^{}.\mathrm{index}(c_{\mathrm{tot}}^{*})$, and $q_{}^{*}=Q(index)$.

## 5. Simulations

In this section, simulations are performed to verify the effectiveness of the proposed method. In the proposed method, the range of *q* can be determined by Lemma 1. Subsequently, given *q*, each width of sub-CBC is equal to H/q. Then, according to Lemma 2, $n_{\max}^{k}$ can be calculated for the *k*th sub-CBC. Given $n\leq n_{\max}^{k}$, a sequence $\left\{c_{s}^{k,1},c_{s}^{k,2},\ldots ,c_{s}^{k,n_{\max}^{k}}\right\}$ can be obtained by using the Formula (10). Thus, $c_{s}^{k}$ is found via the minimum cost principle. Furthermore, the total minimum deployment cost is computed by $\sum_{k=1}^{q}c_{s}^{k}$ for *q* equipartition strategy. 

### 5.1. The Influence of H on the Performance

**Figure 4:**
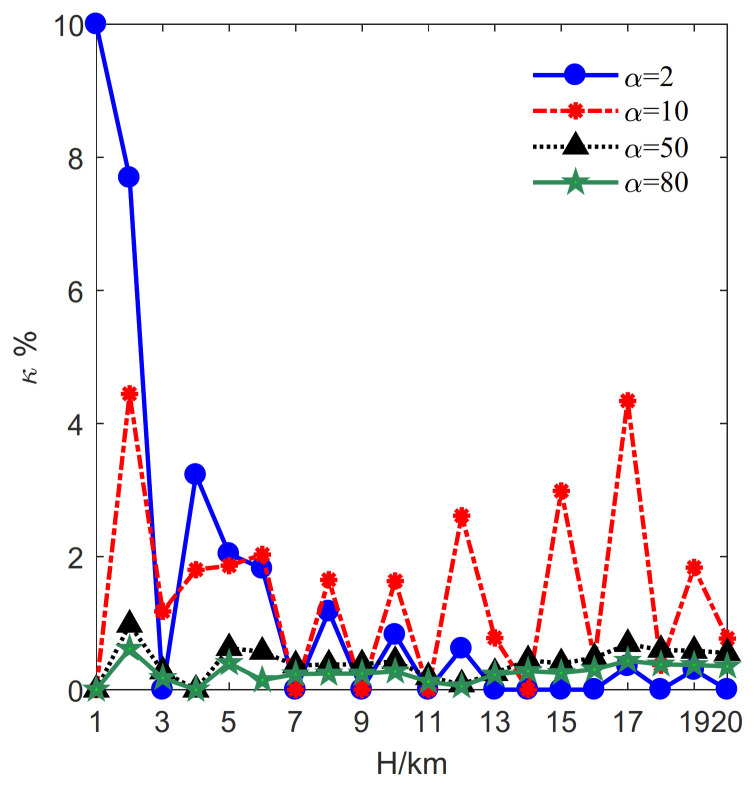
The influence of *H* on the change rates of deployment cost.

Let $l_{\max}=2\;\mathrm{km},\;\mu =0.1\;\mathrm{km}$, $R_{\min}=3\;\mathrm{km}$. *H* varies from 1 km to 20 km with a step size of 1 km. The optimization results for α=2, 10, 50, 80 are plotted in Figure 4.

The change rate κ is exploited to measure the performance achieved by the proposed method and the method mentioned in [33]; κ is defined as follows:

(11) $$\kappa =100\times \left(\tau_{1}-\tau_{2}\right)/\tau_{1}$$

where $\tau_{1}$ and $\tau_{2}$ represents the optimal deployment parameter obtained by [33] and this paper, respectively.

First, the influence of *H* on the minimum deployment cost is investigated. Correspondingly, $\tau_{j},\;j=1,2$ denotes the minimum deployment cost. In this case, the optimal deployment cost change rate revealed by Formula (11) is illustrated in Figure 4.

It can be seen that the value of κ is always larger than or equal to zero when α=2, 10, 50, 80, indicating that the proposed method requires less deployment cost. Specifically, when α=2, 10, the value of κ is much larger. The larger κ is the better cost-saving effect can be achieved. Besides, in most cases, the optimal results under α=2, 10 are superior to those under α=50, 80. Therefore, the proposed method performs better when α=2, 10. Although the change rate decreases in the case of α=50, 80, Figure 4 shows that the proposed method can still obtain less minimum deployment cost.

**Figure 5:**
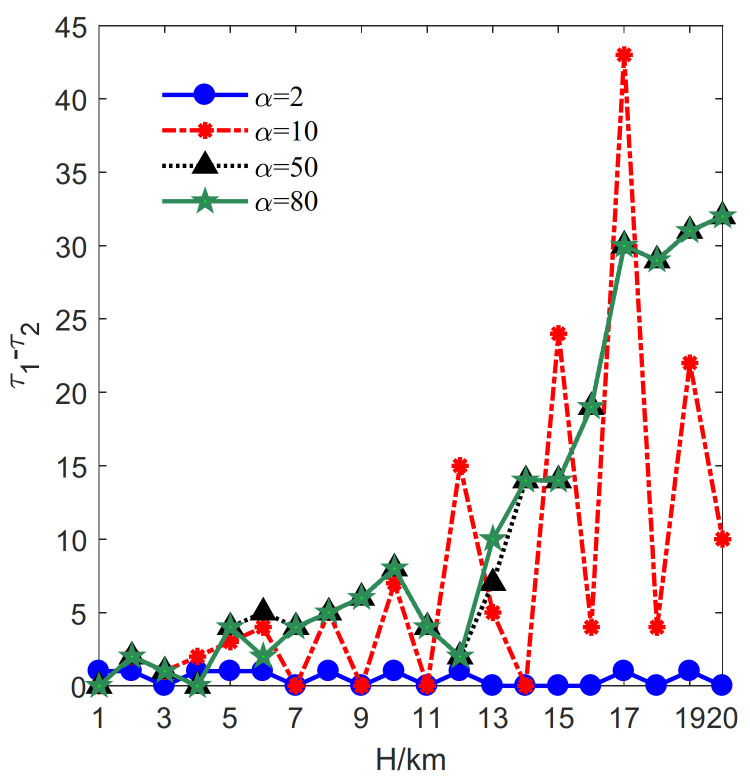
The influence of *H* on the relative changes of deployment cost.

It can be seen from Figure 5 that the magnitude of reduced cost for α=50, 80 has the same level as that of α=2, 10. However, $c_{\mathrm{tot}}^{*}$ is dramatically large for α=50, 80, making the change rate descend.

**Figure 6:**
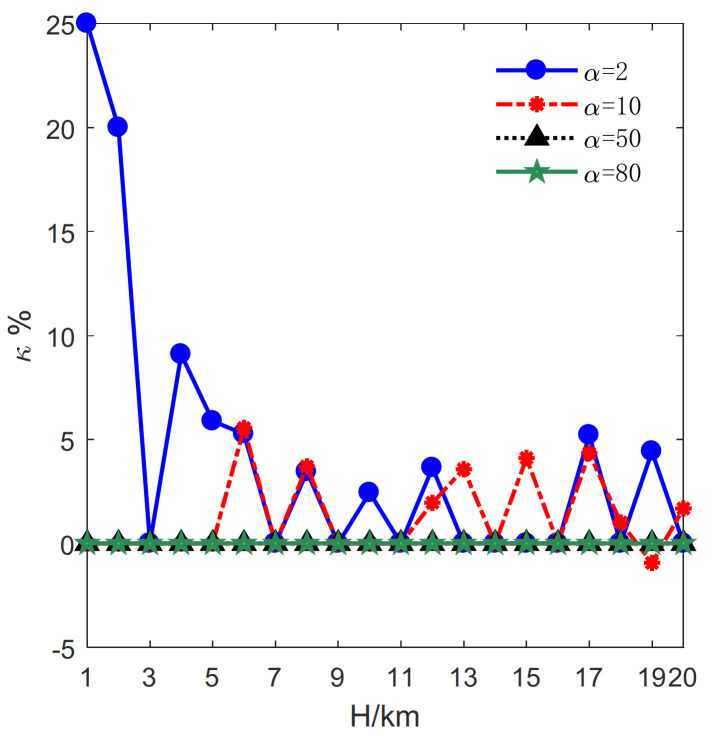
The influence of *H* on the change rates of the number of transmitters.

**Figure 7:**
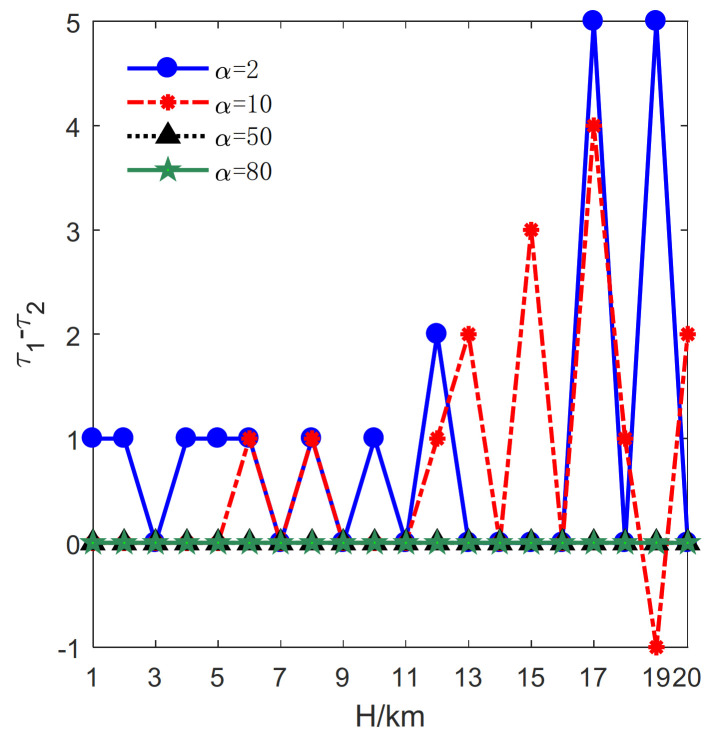
The influence of *H* on the relative change of the number of transmitters.

**Figure 8:**
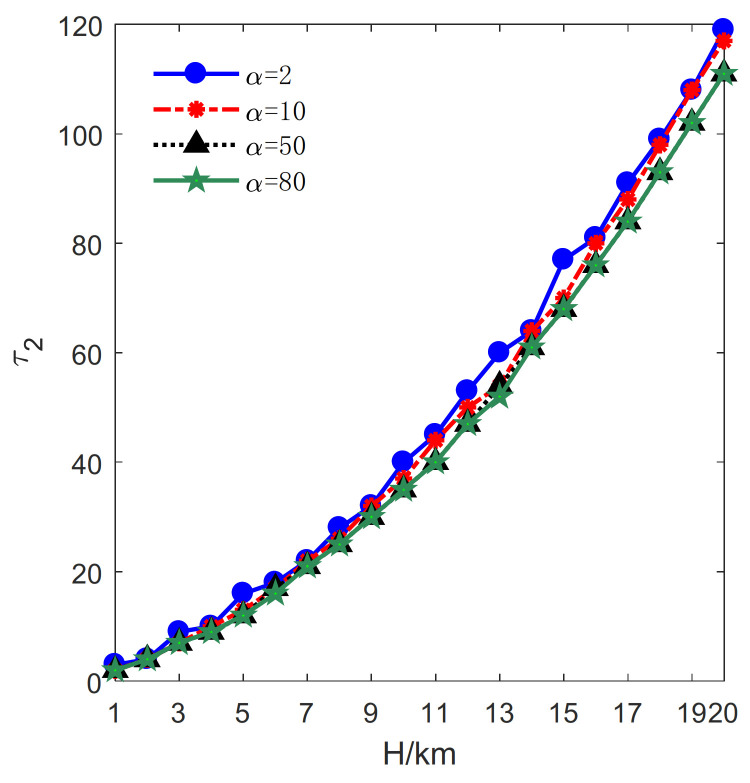
The influence of *H* on the total number of transmitters.

Then, the impact of *H* on the total number of transmitters is evaluated. The results are plotted in Figure 6, Figure 7 and Figure 8. Correspondingly, $\tau_{j},\;j=1,2$ denotes the optimal number of transmitters in Formula (11). The change rate of the number of transmitters is illustrated in Figure 6. The change rates for α=2, 10 are almost larger or equal to zero. By contrast, the change rates are constant and equal to zero for α=50, 80.

Besides, the relative change of the number of transmitters is illustrated in Figure 7. It can be seen from Figure 7 that the relative changes of the numbers of transmitters are equal to zero for α=50, 80 leading the change rates equal to zero. Since a transmitter is far more expensive than a receiver, both methods exploit as few transmitters as possible. Actually, the number of transmitters cannot be further reduced by the proposed method under these conditions.

As illustrated in Figure 6 and Figure 7, though the proposed method exploits one more transmitter and obtains the change rate of −0.93% in the case of α=10, H=19km, the results indicate that the proposed method requires fewer transmitters in most case. 

Additionally, it can be observed from Figure 8 that the number of transmitters increases dramatically when *H* becomes large, leading to a decrease in the change rate. Moreover, a tendency that the number of transmitters decreases with the increase of α is shown in Figure 8. Because of the considerable cost of a transmitter, it is economical to deploy more receivers to pair with one transmitter for extending the PCA. Consequently, the number of transmitters decreases with the number of patterns.

**Figure 9:**
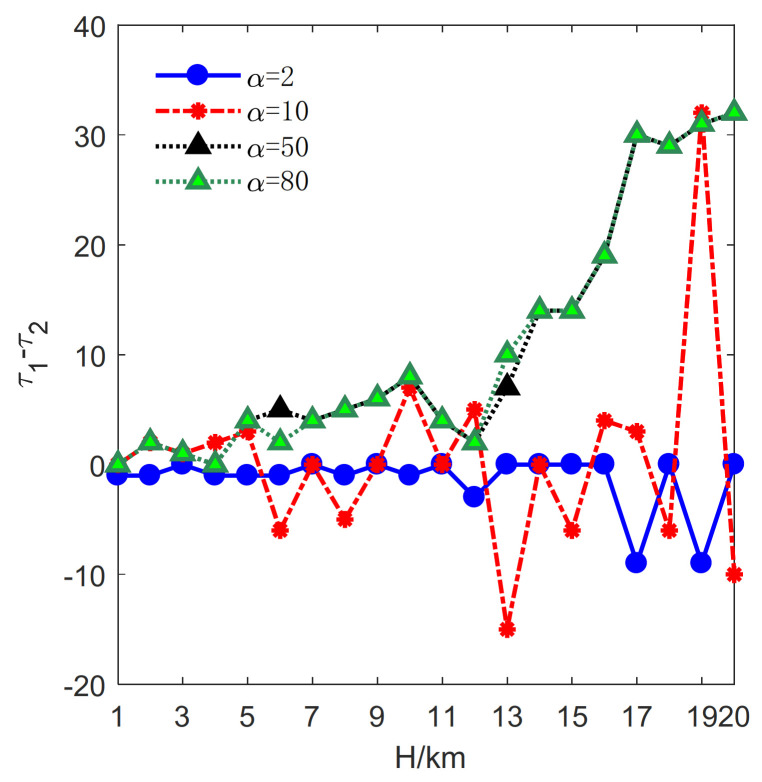
The influence of *H* on the relative change numbers of receivers.

Subsequently, the impact of *H* on the relative change number of receivers is discussed in Figure 9. In this case, $\tau_{1}$ and $\tau_{2}$ represent the optimal number of receivers, respectively.

The relative change number of receivers fluctuates around zero in the condition of α=2, 10, by contrast, the curves are constantly larger than zero in the condition of α=50, 80. The high cost of a transmitter makes it challenging to improve the optimal result on transmitters, but the proposed method can still reduce the optimal result of receivers.

Taking α=50, H=5km as an example, the optimal deployment sequences obtained by the proposed method are $S_{\mathrm{opt}}^{1}=\left\{1,P^{2},2,P^{3}\right\},\;S_{\mathrm{opt}}^{2}=\left\{1,P^{4},3,P^{3}\right\}$ and $S_{\mathrm{opt}}^{3}=\left\{4,P^{4},1,P^{5}\right\}$. Therefore, the total cost of these sequences is 646. By contrast, the optimal deployment sequences obtained by the method in [33] are $S_{\mathrm{opt}}^{1}=\left\{1,P^{2},2,P^{3}\right\},S_{\mathrm{opt}}^{2}=\left\{1,P^{3},3,P^{4}\right\}$ and $S_{\mathrm{opt}}^{3}=\left\{1,P^{3},4,P^{5}\right\}$, and the total cost of these sequences is 642. It is worth emphasizing that there is a large peak for α=10, H=19km. In this condition, although the proposed method exploits one more transmitter, it requires 32 fewer receivers. Therefore, it still reduces the cost.

### 5.2. The Influence of $R_{\min}$ on the Performance

**Figure 10:**
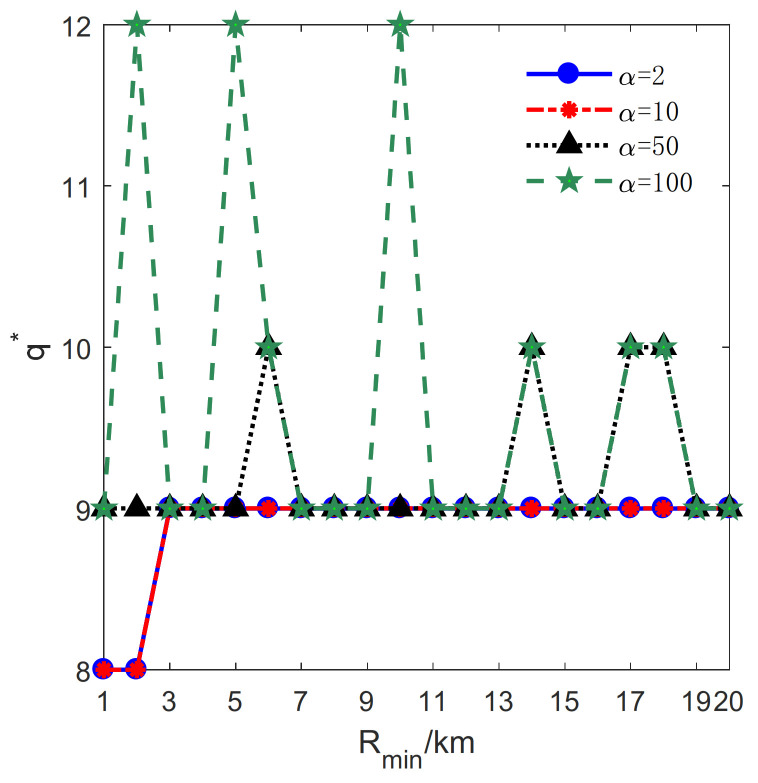
The relationship between the optimal number of sub-circles $q^{*}$ and $R_{\min}$.

The influence of $R_{\min}$ on the proposed method is studied in this section. Let $l_{\max}=2\;\mathrm{km},\;\mu =0.1\;\mathrm{km}$, H=19km. $R_{\min}$ varies from 1 km to 20 km with a step size of 1 km. Besides, *M* and *N* denote the total number of transmitters and receivers, respectively (see Figure 10).

For α=2, 10, the value of $q^{*}$ varies at first and tends to be stable as the $R_{\min}$ increases; for α=50, 100, $q^{*}$ fluctuates as the $R_{\min}$ increases. It reveals that for a small α and a large $R_{\min}$, $q^{*}$ does not change with the increase of $R_{\min}$.

**Figure 11:**
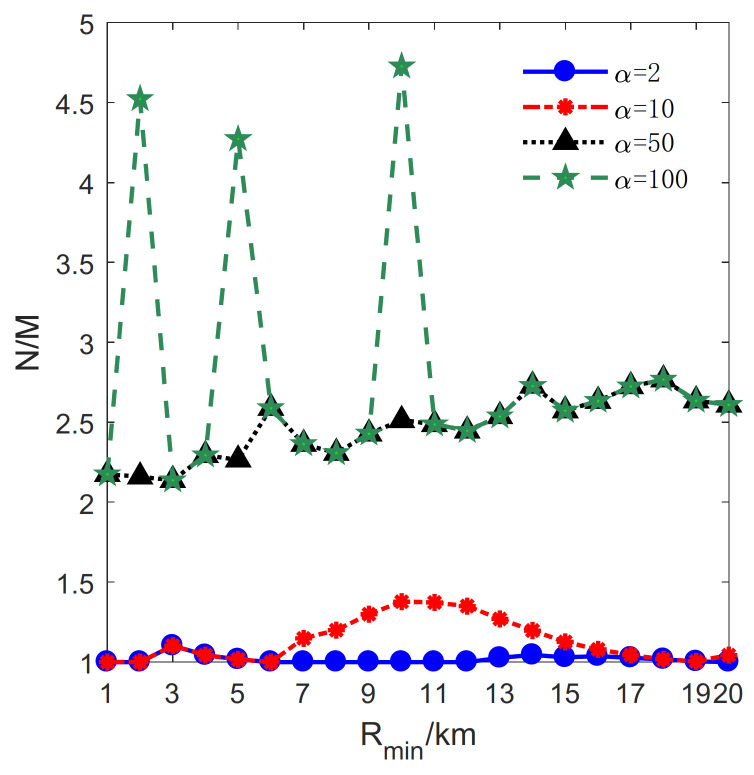
The change of the ratio of the number of transmitters to receivers with the inner radius.

In Figure 11, the relation between $R_{\min}$ and the ratio of *N* to *M* is illustrated. It can be seen that the ratio in the condition of α=50, 100 is almost twice that of α=2, 10. When α becomes larger, the pattern is composed of more receivers. Also, the more receivers contained in a pattern, the narrower barrier coverage is built [32]. Thus, in the case of α=50, 100, the whole area may need to be divided into more sub-circles, resulting in the fluctuation of q*.

Additionally, as depicted in Figure 11, it is worth emphasizing that the number of transmitters is almost equal to that of receivers when α=2. In other words, the $P^{1}$ is mainly used in the deployment. This result is caused by two reasons. One reason is that the first receiver obtains the maximum ECR, according to lemma 3. The other reason is that the difference in the cost between a transmitter and a receiver is little. Therefore, it is economical and efficient to adopt as many *p*^1^ as possible.

**Figure 12:**
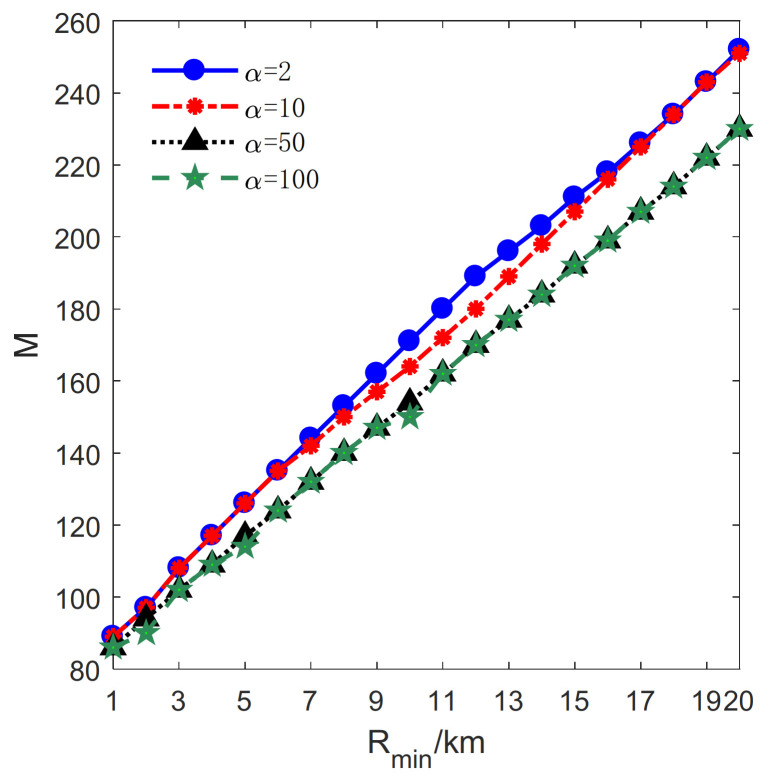
The relationship between the number of transmitters *M* and $R_{\min}$.

Next, the relationship between $R_{\min}$ and *M* is investigated, and the result is shown in Figure 12. It can be seen that the number of transmitters increases approximately linearly with $R_{\min}$ for α=2, 10, 50, 100. According to Formula (5), the increase of $R_{\min}$ leads to an increase in the radius of the deployment curve and a decrease in the PCA. This change means that more transmitters are required as $R_{\min}$ increases. Moreover, given $R_{\min}$, the result in Figure 12 reveals that the number of transmitters decreases as α increases. Thus, it is worth deploying more receivers instead of a transmitter because the receivers are highly cheap when α=50, 100.

**Figure 13:**
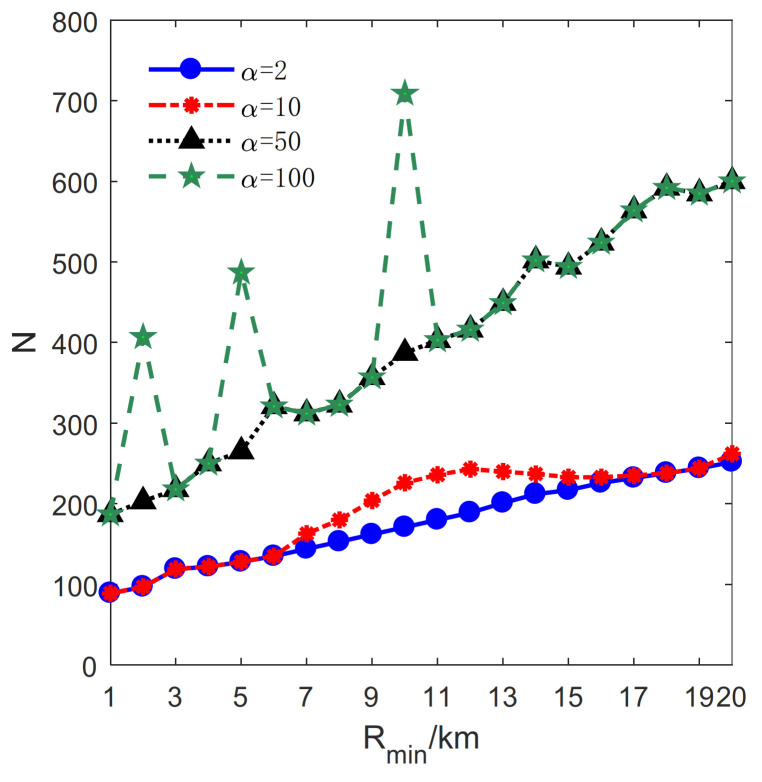
The relationship between the number of receivers *N* and $R_{\min}$.

Furthermore, the relationship between $R_{\min}$ and *N* is studied, and the result is illustrated in Figure 13. The number of receivers increases with $R_{\min}$ for α=2, 10, 50, 100. It should be noted that there are three peaks for α=100, H=2,5,10. It is because more sub-circles are divided in these cases, as plotted in Figure 10. Moreover, it is noteworthy that *N* increases significantly when α=10, 7≤H≤12 and then tends to be stable as the $R_{\min}$ increases. It can be explained by the slope of the transmitter numbers that decrease in this condition, as depicted in Figure 12. The decreased slope indicates that the magnitude of the increased number of transmitters decreases. To enlarge the PCA, more receivers are exploited in the patterns.

## 6. Conclusions

A deployment optimization method is proposed in this paper to construct circular barrier coverage with an equipartition strategy. The concepts of multistatic radar coverage and CBC are introduced. Based on the equipartition strategy, a circular FoI is equally divided into *q* sub-circles. Each sub-circle is blanketed by a sub-CBC composed of several multistatic radar deployment patterns. Moreover, the optimization conditions for determining the optimal deployment patterns in a sub-CBC are studied. The conditions reveal that at most two varied types of patterns are needed by an optimal deployment sequence. Besides, a model based on minimum deployment cost is proposed to optimize the deployment on the whole circular FoI. The model is divided into two sub-models, and a two-stage optimization algorithm is proposed to solve the model. In the inner model, it is assumed that the width of a sub-circle is given. Based on the optimization conditions, ILP and EM are jointly exploited to obtain the types and numbers of deployment patterns. Besides, a novel formula is derived to improve the result of the maximum number of receivers in a pattern and reduce the search scope of EM. In the outer model, the improved formula is proposed to determine the number range of sub-circles and the width of a sub-circle. Then, EM is adopted to determine the minimum deployment cost and the optimal deployment patterns in each sub-CBC. Finally, simulations are conducted to validate the effectiveness of the proposed method. It is worth mentioning that the equipartition strategy results in a locally optimal solution. To improve the optimization results, the non-equipartition strategy should be considered in the future. Moreover, there are many overlapping areas in CBC, making it challenging to improve the coverage effectiveness. Therefore, it is beneficial to explore new coverage models, such as the regular polygonal model.

## Figures and Tables

**Figure A1:**
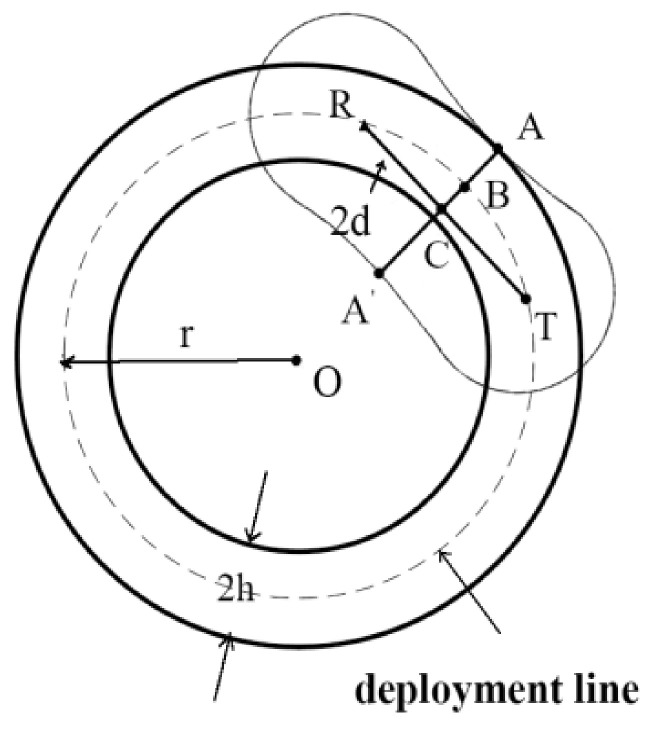
Schematic diagram of the upper limit of the sub-circular barrier width.

## Data Availability

Not applicable.

## Appendix A

**Proof of** **Lemma 1.** As shown in Figure A1, the line AA′ is the perpendicular bisector of the line TR. Let B denotes the intersection point of the line AA′ and the deployment curve. Let C denotes the intersection point of line AA′ and line TR. The distance between the first pair of transmitters and receivers is 2*d*, the radius of the deployment curve is *r*. Clearly, |BC|=r−|OC|, $|\mathrm{OC}|=\sqrt{r^{2}-d_{}^{2}}$, $|\mathrm{BC}|=r-\sqrt{r^{2}-d_{}^{2}}$. Besides, since h=|AC|−|BC| and $|\mathrm{AC}|=\sqrt{l_{\max}^{2}-d^{2}}$, thus we have

(A1) $$h=\sqrt{l_{\max}^{2}-d^{2}}+\sqrt{r^{2}-d_{}^{2}}-r$$

It is easy to know that *h* is a monotonically decreasing function concerning *d*. Let $h_{\max}$ denotes maximum width. Additionally, waist-liked area means $d_{1}\geq l_{\max}/\sqrt{2}$. According to Formula (A1), we obtain $h_{\max}=l_{\max}/\sqrt{2}+\sqrt{r^{2}-0.5l_{\max}^{2}}-r$. Furthermore, $h_{\max}$ increases as *r* increases, and the innermost CBC obtains the smallest radius of $R_{\min}+h$. Therefore, in width-equipartition strategy, substituting *r* with $R_{\min}+h$, then $h_{\sup}$ is calculated as follows:

$$h_{\sup}=\frac{1}{3}(\sqrt{2}l_{\max}+\sqrt{R_{\min}^{2}+\sqrt{2}R_{\min}l_{\max}-l_{\max}^{2}}-R_{\min})$$

The proof of Lemma 1 is completed.  □

By contrast, in [33], it claims the width range of CBC is $\left(0,\sqrt{2}l_{\max}^{}\right]$. However, $\sqrt{2}l_{\max}^{}$ is the maximum value of the $\left|AA\prime \right|$. When $2h=\sqrt{2}l_{\max}^{}$ it means points C and B are coincident, which is impossible in this scenario.

**Proof of** **Lemma 2.** (1) In the beginning, $h>\sqrt{r^{2}+l_{\max}^{2}}-r$ means ${\left(2r+h\right)}^{2}h^{2}-l_{\max}^{4}>0$. To guarantee $R_{k},\;k=1,2,\ldots ,\left\lceil \left(n+1\right)/2\right\rceil$ is valid, $\theta_{k}>0$ should hold. According to Formula (3), we obtain

(A2) $$\mathrm{acos}\{[(r^{2}+R^{2})\cos(\sum_{m=1}^{k-1}\theta_{m})-\sqrt{{\left(R^{2}-r^{2}\right)}^{2}(\cos^{2}(\sum_{m=1}^{k-1}\theta_{m})-1)+l_{\max}^{4}})]/2rR\}>\sum_{m=1}^{k-1}\theta_{m}$$

(A3) $${\left(R^{2}-r^{2}\right)}^{2}(\cos^{2}(\sum_{m=1}^{k-1}\theta_{m})-1)+l_{\max}^{4}\geq 0$$

According to Formula (A2), we obtain

$$(r^{2}+R^{2})\cos(\sum_{m=1}^{k-1}\theta_{m})-\sqrt{{\left(R^{2}-r^{2}\right)}^{2}(\cos^{2}(\sum_{m=1}^{k-1}\theta_{m})-1)+l_{\max}^{4}})<2rR\cos(\sum_{m=1}^{k-1}\theta_{m})\;$$

Furthermore, ${\left(R-r\right)}^{2}\cos(\sum_{m=1}^{k-1}\theta_{m})<\sqrt{{\left(R^{2}-r^{2}\right)}^{2}(\cos^{2}(\sum_{m=1}^{k-1}\theta_{m})-1)+l_{\max}^{4}}$. From the inequality above, we obtain

(A4) $$\cos^{2}(\sum_{m=1}^{k-1}\theta_{m})>\frac{{\left(R^{2}-r^{2}\right)}^{2}-l_{\max}^{4}}{4rR{\left(R-r\right)}^{2}}$$

Note that R=r+h is the outer radius of a sub-circular barrier, and the cosine function decreases in the first quadrant. Thus we obtain

(A5) $$\sum_{m=1}^{k-1}\theta_{m}<\mathrm{acos}(\sqrt{\frac{{\left(2r+h\right)}^{2}h^{2}-l_{\max}^{4}}{4r(r+h)h^{2}}})$$

Besides, according to Formula (A3), we obtain:

(A6) $$\sum_{m=1}^{k-1}\theta_{m}\leq \mathrm{acos}(\sqrt{\frac{{\left(2r+h\right)}^{2}h^{2}-l_{\max}^{4}}{{\left(2r+h\right)}^{2}h^{2}}})$$

Clearly, ${\left(2r+h\right)}^{2}h^{2}>4r(r+h)h^{2}$. Combining Formulas (A5) and (A6), we achieve

$$\sum_{m=1}^{k-1}\theta_{m}<\mathrm{acos}(\sqrt{\frac{{\left(2r+h\right)}^{2}h^{2}-l_{\max}^{4}}{4r(r+h)h^{2}}}).$$

Particularly, for $k=\left\lceil \left(n+1\right)/2\right\rceil$, we obtain $\sum_{m=1}^{\left\lceil (n+1)/2\right\rceil -1}\theta_{m}<\mathrm{acos}(\sqrt{\frac{{\left(2r+h\right)}^{2}h^{2}-l_{\max}^{4}}{4r(r+h)h^{2}}})$. Thus $n_{\max}=2u+1$. Next, it is desirable to prove $\cos(\sum_{m=1}^{u+1}\theta_{m})>0$ is the sufficient and necessary condition for $2\sum_{m=1}^{u+1}\theta_{m}<\pi$. Since $\cos(\sum_{m=1}^{u+1}\theta_{m})>0$, according to Formula (4), we obtain

$$(r^{2}+R^{2})\cos(\sum_{m=1}^{u}\theta_{m})>\sqrt{{\left(R^{2}-r^{2}\right)}^{2}(\cos^{2}(\sum_{m=1}^{u}\theta_{m})-1)+l_{\max}^{4}}).$$

With the algebraic operation, we obtain

(A7) $$\cos^{2}(\sum_{m=1}^{u}\theta_{m})>\frac{l_{\max}^{4}-{\left(R^{2}-r^{2}\right)}^{2}}{4r^{2}R^{2}}$$

Note that $l_{\max}^{4}-{\left(R^{2}-r^{2}\right)}^{2}<0$, therefore, Formula (A7) holds. It means $2\sum_{m=1}^{u+1}\theta_{m}<\pi$ holds (2) When $\max\{l_{\max}^{}-2r,0\}<h\leq \min\{\sqrt{r^{2}+l_{\max}^{2}}-r,h_{\sup}\}$, we obtain ${\left(2r+h\right)}^{2}h^{2}-l_{\max}^{4}\leq 0$. In this case, both Formulas (A2) and (A3) hold. In other words, for $P^{n}$, *n* is a valid number as long as $\sum_{m=1}^{t}2\theta_{m}\leq \pi ,$ $t=\left\lceil n/2\right\rceil$ holds. Nevertheless, due to the CBC, we have $\sum_{m=1}^{u}2\theta_{m}\leq \pi ,\;u\leq \left\lceil n/2\right\rceil$. And according to Formula (4), with the algebraic operation, we obtain $\left(r^{2}+R^{2})\cos(\sum_{m=1}^{u-1}\theta_{m})\geq \sqrt{{\left(R^{2}-r^{2}\right)}^{2}(\cos^{2}(\sum_{m=1}^{u-1}\theta_{m})-1)+l_{\max}^{4}}\right)$. Furthermore, we have $\sum_{m=1}^{u-1}\theta_{m}\leq \mathrm{acos}(\sqrt{\frac{l_{\max}^{4}-{\left(R^{2}-r^{2}\right)}^{2}}{4r^{2}R^{2}}})$. Substituting *u* with $\left\lceil n/2\right\rceil$, we obtain $\sum_{m=1}^{\left\lceil n/2\right\rceil -1}\theta_{m}\leq \mathrm{acos}(\sqrt{\frac{l_{\max}^{4}-{\left(R^{2}-r^{2}\right)}^{2}}{4r^{2}R^{2}}})\;$ and $n_{\max}=2(u+1)$. The proof of Lemma 2 is completed.  □

**Proof** **of Lemma 3.** First, if k=1 we obtain $\sum_{m=1}^{k-1}\theta_{m}=0$. According to Formula (A4), we have:

$$2rR\cos(\sum_{m=1}^{k}\theta_{m})=(r^{2}+R^{2})\cos(\sum_{m=1}^{k-1}\theta_{m})-\sqrt{{\left(R^{2}-r^{2}\right)}^{2}(\cos^{2}(\sum_{m=1}^{k-1}\theta_{m})-1)+l_{\max}^{4}}.$$

With the algebraic operation, we have

$$2rR\left(\cos\sum_{m=1}^{k}\theta_{m}-\cos\sum_{m=1}^{k-1}\theta_{m}\right)={\left(r-R\right)}^{2}\cos\sum_{m=1}^{k-1}\theta_{m}-\sqrt{{\left(R^{2}-r^{2}\right)}^{2}(\cos^{2}(\sum_{m=1}^{k-1}\theta_{m})-1)+l_{\max}^{4}}$$

Applying the Lagrange Mean Value Theorem, we obtain

(A8) $$2rR\theta_{k}\sin\xi_{k}=\sqrt{{\left(R^{2}-r^{2}\right)}^{2}(\cos^{2}(\sum_{m=1}^{k-1}\theta_{m})-1)+l_{\max}^{4}}-{\left(R-r\right)}^{2}\cos(\sum_{m=1}^{k-1}\theta_{m})$$

where $\xi_{k}$ satisfies

(A9) $$\sum_{m=1}^{k-1}\theta_{m}<\xi_{k}<\sum_{m=1}^{k}\theta_{m}$$

Let $f(x)=\sqrt{{\left(R^{2}-r^{2}\right)}^{2}(\cos^{2}x-1)+l_{\max}^{4}}-{\left(R-r\right)}^{2}\cos x$, 0<x<φ. Then,

$$f^{\prime }(x)={\left(R-r\right)}^{2}\left(1-\frac{{\left(R+r\right)}^{2}\cos x}{\sqrt{{\left(R^{2}-r^{2}\right)}^{2}(\cos^{2}x-1)+l_{\max}^{4}}}\right)\sin x$$

Note that the formula ${\left(R+r\right)}^{2}\cos x>\sqrt{{\left(R^{2}-r^{2}\right)}^{2}(\cos^{2}x-1)+l_{\max}^{4}}$ can be rewritten as:

(A10) $$4rR{\left(R+r\right)}^{2}\cos^{2}x>l_{\max}^{4}-{\left(R^{2}-r^{2}\right)}^{2}$$

If $h>\sqrt{r^{2}+l_{\max}^{2}}-r$, then we obtain $l_{\max}^{4}-{\left(R^{2}-r^{2}\right)}^{2}<0$. It leads Formula (A10) holds. By contrast, if $h\leq \sqrt{r^{2}+L_{\max}^{2}}-r$, then we obtain $l_{\max}^{4}-{\left(R^{2}-r^{2}\right)}^{2}\geq 0$. Furthermore, Formula (A10) can be rewritten as:

$$x\leq \mathrm{acos}(\sqrt{\frac{l_{\max}^{4}-{\left(R^{2}-r^{2}\right)}^{2}}{4rR{\left(r+R\right)}^{2}}})$$

Since R=r+h, then we obtain: $\mathrm{acos}(\sqrt{\frac{l_{\max}^{4}-{\left(2r+h\right)}^{2}h^{2}}{4r^{2}{\left(r+h\right)}^{2}}})\leq \mathrm{acos}(\sqrt{\frac{l_{\max}^{4}-{\left(2r+h\right)}^{2}h^{2}}{4rR{\left(r+R\right)}^{2}}})$. Therefore, if 0<x<φ, then $f^{\prime }(x)<0$. It reveals that the right part of the equation in Formula (A8) is monotonically decreasing. Besides, according to Formula (A9), the sequence $\left\{\xi_{k},k=1,2,\ldots .u\right\}$ is strictly monotonically increasing. Also, it is well-known that the sine function is the monotonic increasing at (0,π/2). Thus, it is safe to claim that the NCA sequence is strictly monotonically decreasing. The proof of Lemma 3 is completed.  □

**Proof of** **Lemma 4.** (1) Since $n_{1}+n_{2}$ is even, then it can be divided into two cases: (a) both $n_{1}$ and $n_{2}$ are even; (b) both $n_{1}$ and $n_{2}$ are odd. Without loss of generality, let $n_{1}=v-i,\;n_{2}=v+i$. (a) When both $n_{1}$ and $n_{2}$ are even, there are two sub-cases: ① if v is even, and then we obtain i is even. According to Formula (5), we obtain

$$\begin{array}{l} 2\omega_{P}(v)=\sum_{k=1}^{v/2}8\theta_{k}+4\theta_{\frac{v}{2}+1}=\sum_{k=1}^{\frac{v+i}{2}}4\theta_{k}-\sum_{k=\frac{v}{2}+1}^{\frac{v+i}{2}}4\theta_{k}+\sum_{k=1}^{\frac{v-i}{2}}4\theta_{k}+\sum_{k=\frac{v-i}{2}+1}^{\frac{v}{2}}4\theta_{k}+4\theta_{\frac{v}{2}+1}\\ =\omega_{P}(n_{1})+\omega_{P}(n_{2})+4\sum_{k=1}^{\frac{i}{2}-1}(\theta_{k+\frac{v-i}{2}}-\theta_{k+\frac{v}{2}})+2(\theta_{\frac{v-i}{2}+1}-\theta_{\frac{v+i}{2}+1}) \end{array}$$

Then

(A11) $$\omega_{P}(n_{1})+\omega_{P}(n_{2})=2\omega_{P}(v)-4\sum_{k=1}^{\frac{i}{2}-1}(\theta_{k+\frac{v-i}{2}}-\theta_{k+\frac{v}{2}})-2(\theta_{\frac{v-i}{2}}-\theta_{\frac{v+i}{2}})$$

Based on Lemma 3 and Formula (A11), we obtain $\omega_{P}(n_{1})+\omega_{P}(n_{2})\leq 2\omega_{P}(v)$. Furthermore, if and only if i=0 the equation holds. And i=0 means $n_{1}=n_{2}$. ② If v is odd, in this case, i is odd. According to Formula (5), we obtain

(A12) $$\begin{array}{l} 2\omega_{P}(v)=8\sum_{k=1}^{(v+1)/2}\theta_{k}=4\sum_{k=1}^{\frac{v+i}{2}}\theta_{k}-4\sum_{k=\frac{v+1}{2}+1}^{\frac{v+i}{2}}\theta_{k}+4\sum_{k=1}^{\frac{v-i}{2}}\theta_{k}+4\sum_{k=\frac{v-i}{2}+1}^{\frac{v+1}{2}}\theta_{k}\\ =\omega_{P}(n_{1})+\omega_{P}(n_{2})+4\sum_{k=1}^{\frac{i-1}{2}}(\theta_{k+\frac{v-i}{2}+1}-\theta_{k+\frac{v+1}{2}})+2(\theta_{\frac{v-i}{2}+1}-\theta_{\frac{v+i}{2}+1}) \end{array}$$

Based on Lemma 3 and Formula (A12), we obtain $\omega_{P}(n_{1})+\omega_{P}(n_{2})<$ $2\omega_{P}(v)$. (b) When both $n_{1}$ and $n_{2}$ are odd, also, there are two sub-cases: ① v is even; ② v is odd. First, for v is even, we obtain $2\omega_{P}(v)=\omega_{P}(n_{1})+\omega_{P}(n_{2})+4\sum_{k=1}^{\frac{i-1}{2}}(\theta_{k+\frac{v-i+1}{2}}-\theta_{k+\frac{v}{2}+1})$. Furthermore, $\omega_{P}(n_{1})+\omega_{P}(n_{2})\leq 2\omega_{P}(v)$. If and only if i=1 the equation holds. And, i=1 means $n_{1},n_{2}$ are consecutive odd. Second, for v is odd, we obtain $\omega_{P}(n_{1})+\omega_{P}(n_{2})=2\omega_{P}(v)-4\sum_{k=1}^{\frac{i}{2}}(\theta_{k+\frac{v-i+1}{2}}-\theta_{k+\frac{v+1}{2}})$ leading $\omega_{P}(n_{1})+\omega_{P}(n_{2})\leq 2\omega_{P}(v)$. If and only if i=0, the equation holds. And i=0 means $n_{1}=n_{2}$. (2) Without loss of generality, it is assumed that $n_{2}=v+i$, $n_{1}=v+1-i$. And let $n_{1}$ is odd, $n_{2}$ is even. Since $n_{1}+n_{2}$ is odd, there are two cases: ① v is odd; ② v is even. First, if v is odd, thus *i* is odd. According to Formula (5), it can be derived that

$$\begin{array}{l} \omega_{P}(v)+\omega_{P}(v+1)=\sum_{k=1}^{\frac{v+i}{2}}4\theta_{k}-\sum_{k=\frac{v+1}{2}+1}^{\frac{v+i}{2}}4\theta_{k}+\sum_{k=1}^{\frac{v+2-i}{2}}4\theta_{k}+\sum_{k=\frac{v+2-i}{2}+1}^{\frac{v+1}{2}}4\theta_{k}\\ +2\theta_{\frac{v+1}{2}+1}=\omega_{P}(n_{1})+\omega_{P}(n_{2})+4\sum_{k=1}^{\frac{i-1}{2}}(\theta_{k+\frac{v+2-i}{2}}-\theta_{k+\frac{v+1}{2}})+2(\theta_{\frac{v+1}{2}+1}-\theta_{\frac{v+i}{2}+1}) \end{array}$$

Then

(A13) $$\omega_{P}(n_{1})+\omega_{P}(n_{2})=\omega_{P}(v)+\omega_{P}(v+1)-2(\theta_{\frac{v+1}{2}+1}-\theta_{\frac{v+i}{2}+1})-4\sum_{k=1}^{\frac{i-1}{2}}(\theta_{k+\frac{v+2-i}{2}}-\theta_{k+\frac{v+1}{2}})$$

According to Formula (A13), we obtain $\omega_{P}(n_{1})+\omega_{P}(n_{2})\leq \omega_{P}(v)+\omega_{P}(v+1)$. If and only if i=1 the equation holds. And, i=1 means $n_{2}=v+1,\;n_{1}=v$. Second, if v is even, in this case, i is even. Similarly, we obtain.

$$\begin{array}{l} \omega_{P}(v)+\omega_{P}(v+1)=\sum_{k=1}^{\frac{v+i}{2}}4\theta_{k}-\sum_{k=\frac{v}{2}+1}^{\frac{v+i}{2}}4\theta_{k}+\sum_{k=1}^{\frac{v+2-i}{2}}4\theta_{k}+\sum_{k=\frac{v+2-i}{2}+1}^{\frac{v+2}{2}}4\theta_{k}\\ +2\theta_{\frac{v}{2}+1}=\omega_{P}(n_{1})+\omega_{P}(n_{2})+4\sum_{k=1}^{\frac{i}{2}}(\theta_{k+\frac{v+2-i}{2}}-\theta_{k+\frac{v}{2}})+2(\theta_{\frac{v}{2}+1}-\theta_{\frac{v+i}{2}+1}) \end{array}$$

Then,

(A14) $$\omega_{P}(n_{1})+\omega_{P}(n_{2})=\omega_{P}(v)+\omega_{P}(v+1)-4\sum_{k=1}^{\frac{i}{2}}(\theta_{k+\frac{v+2-i}{2}}-\theta_{k+\frac{v}{2}})-2(\theta_{\frac{v}{2}+1}-\theta_{\frac{v+i}{2}+1})$$

According to Formula (A14), we obtain $\omega_{P}(n_{1})+\omega_{P}(n_{2})\leq \omega_{P}(v)+\omega_{P}(v+1)$. If and only if i=0, the equation holds. And, i=0 means $n_{2}=v,\;n_{1}=v+1$. The proof of Lemma 4 is completed.  □
